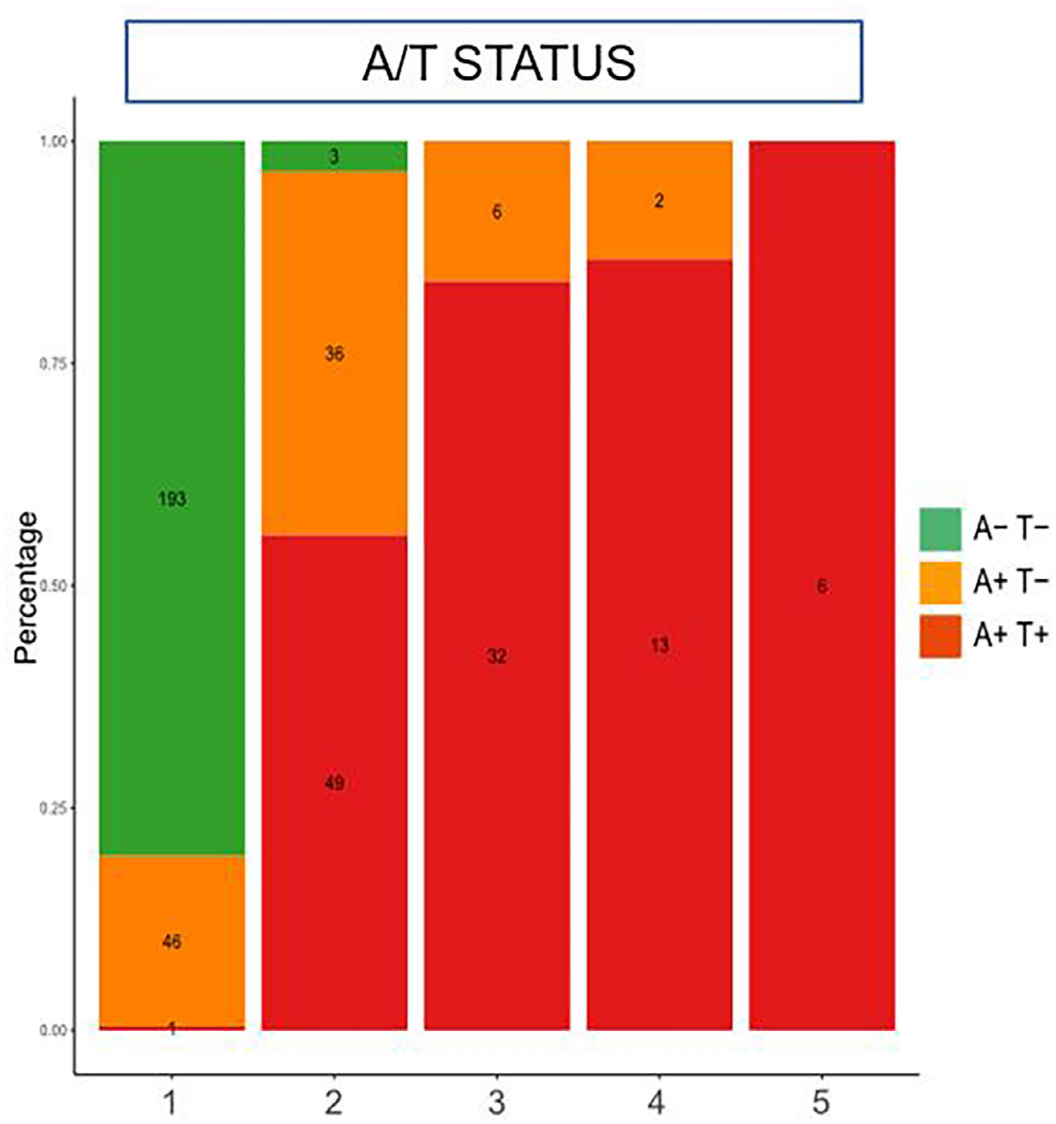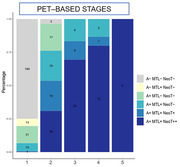# Plasma Tau Biomarkers for Alzheimer's Disease Staging

**DOI:** 10.1002/alz.088408

**Published:** 2025-01-09

**Authors:** Laia Montoliu‐Gaya, Gemma Salvadó, Nicholas J. Ashton, Shorena Janelidze, Johanna Nilsson, Niklas Mattsson‐Carlgren, Sophia Weiner, Sebastian Palmqvist, Juan Lantero Rodriguez, Gunnar Brinkmalm, Erik Stomrud, Henrik Zetterberg, Johan Gobom, Kaj Blennow, Oskar Hansson

**Affiliations:** ^1^ Institute of Neuroscience and Physiology, Department of Psychiatry and Neurochemistry, University of Gothenburg, Mölndal Sweden; ^2^ Clinical Memory Research Unit, Lund University, Lund Sweden; ^3^ University of Gothenburg, Mölndal Sweden; ^4^ Clinical Memory Research Unit, Department of Clinical Sciences, Lund University, Lund Sweden; ^5^ Department of Psychiatry and Neurochemistry, Institute of Neuroscience and Physiology, The Sahlgrenska Academy, University of Gothenburg, Mölndal, Gothenburg Sweden; ^6^ Clinical Memory Research Unit, Department of Clinical Sciences Malmö, Faculty of Medicine, Lund University, Lund Sweden; ^7^ Department of Psychiatry and Neurochemistry, Institute of Neuroscience and Physiology, The Sahlgrenska Academy, University of Gothenburg, Mölndal Sweden; ^8^ Clinical Memory Research Unit, Department of Clinical Sciences Malmö, Faculty of Medicine, Lund University, Lund, Sweden, Malmö Sweden; ^9^ Department of Psychiatry and Neurochemistry, Institute of Neuroscience and Physiology, the Sahlgrenska Academy at the University of Gothenburg, Mölndal Sweden; ^10^ Clinical Neurochemistry Laboratory, Sahlgrenska University Hospital, Gothenburg, VGR Sweden; ^11^ Institute of Neuroscience and Physiology, The Sahlgrenska Academy at the University of Gothenburg, Mölndal Sweden; ^12^ Clinical Memory Research Unit, Department of Clinical Sciences, Lund University, and Memory Clinic, Skåne University Hospital, Malmö Sweden

## Abstract

**Background:**

Plasma phosphorylated tau (p‐tau), particularly p‐tau217, has demonstrated its reliability as a biomarker for diagnosing Alzheimer's disease (AD). Coming challenges in the field include determining whether quantifying additional plasma phosphorylated, and non‐phosphorylated tau species could enhance diagnostic accuracy, prognosis, and improve patient monitoring by effectively staging the disease.

**Method:**

We used a mass spectrometric targeted method to simultaneously quantify the levels of six different phosphorylated (p‐tau 181, 199, 202, 205, 217 and 231), and six non‐phosphorylated (0N CNS‐specific, 1N CNS‐specific, tau195‐209, tau212‐221, PNS‐specific 7‐14, PNS‐specific 151‐167) tau peptides in plasma. We analyzed a total of 553 samples from participants in the BioFINDER‐2 cohort, with available amyloid‐PET ([^18^F]flutemetamol, n=358) and/or tau‐PET ([^18^F]RO‐948, n=458). We studied how plasma levels were increased in participants based on amyloid‐ and tau‐PET status (A‐/A+, T‐/T+) and PET stages based on amyloid positivity and tau positivity in early (medial temporal lobe [MTL]) and intermediate regions (neotemporal [NeoT]).

**Result:**

Visual inspection of locally estimated scatterplot smoothing (LOESS) analyses showed that the levels of specific plasma tau markers became abnormal (z‐score>2, >95%CI of CU‐) at different stages in relation to Tau‐PET SUVR (ordered: p‐tau217, p‐tau205, 0N CNS‐specific, and tau212‐221). We then created a 5‐step disease staging model based on the positivity for these biomarkers and compared them with stages based on amyloid and tau PET status (Figure 1) and PET‐based stages (Figure 2). In stage‐1 (negative for all plasma biomarkers), 80.8% of participants were A‐T‐ and 19.2% were A+T‐. In stage‐2 (p‐tau217+), only 3.4% were A‐T‐, 40.9% were A+T‐ and 55.7% were A+T+. Specifically, 22.6% were MTL+NeoT‐, 22.6% MTL+NeoT+, and 31% MTL+NeoT++. In stages‐3 and ‐4 (p‐tau217+ p‐tau205+, and p‐tau217+ p‐tau205+ 0N CNS‐specific+) all participants were A+MTL+, and 83.3%‐86.7% were also NeoT+ or NeoT++. Finally, in stage‐5 (all markers positive), 100% of the participants were A+T+ and they all were A+MTL+NeoT++.

**Conclusion:**

Plasma tau species exhibit sequential changes, and our data supports the notion of staging AD using fluid biomarkers. The stages identified in plasma may not necessarily correspond to PET imaging stages. Future analyses will focus on determining the utility of plasma‐based staging model for prognosis.